# Cancer-on-chip: a 3D model for the study of the tumor microenvironment

**DOI:** 10.1186/s13036-023-00372-6

**Published:** 2023-08-17

**Authors:** Elisa Cauli, Michela Anna Polidoro, Simona Marzorati, Claudio Bernardi, Marco Rasponi, Ana Lleo

**Affiliations:** 1 Department of Electronics, Information and Bioengineering, Politecnico Di Milano, Milan, Italy; 2 Accelera Srl, Nerviano, Milan, Italy; 3 Hepatobiliary Immunopathology Laboratory, IRCCS Humanitas Research Hospital, Rozzano, Milan, Italy; 4 Department of Biomedical Sciences, Humanitas University, Pieve Emanuele, Milan, Italy; 5 Division of Internal Medicine and Hepatology, Department of Gastroenterology, IRCCS Humanitas Research Hospital, Rozzano, Milan, Italy

**Keywords:** Cancer-on-chip, Tumor microenvironment, Metastasis, Organ-on-chip, Microfluidics, Pre-clinical models

## Abstract

The approval of anticancer therapeutic strategies is still slowed down by the lack of models able to faithfully reproduce in vivo cancer physiology. On one hand, the conventional in vitro models fail to recapitulate the organ and tissue structures, the fluid flows, and the mechanical stimuli characterizing the human body compartments. On the other hand, in vivo animal models cannot reproduce the typical human tumor microenvironment, essential to study cancer behavior and progression. This study reviews the cancer-on-chips as one of the most promising tools to model and investigate the tumor microenvironment and metastasis. We also described how cancer-on-chip devices have been developed and implemented to study the most common primary cancers and their metastatic sites. Pros and cons of this technology are then discussed highlighting the future challenges to close the gap between the pre-clinical and clinical studies and accelerate the approval of new anticancer therapies in humans.

## Background

Cancer is the main disease burden worldwide. An estimated 26.3% increase in incident cancer cases and a 20.9% one in cancer deaths have occurred since 2010. Significant growth is expected in the next two decades [1]. Prevention actions need to be defined and implemented, especially in low-to-middle-income countries [2]. However, much effort is constantly deployed to find successful therapeutic solutions to this widespread disease [3–5]. Notwithstanding, the lack of models reproducing the in vivo cancer physiology represents one of the main problems in the development of anti-cancer therapeutic strategies. Indeed, the limits of the currently used in vitro and in vivo models for tumor studies hampered the thorough understanding of its behavior and the underlying molecular mechanisms [6]. Cancer two-dimensional (2D) in vitro models are routinely used due to their easy application, low cost, and well-established procedures to perform cancer studies. However, their main limitation relies on the impossibility of correctly reproducing the three-dimensional (3D) structure of the human tumor niche, the complex interactions between tumor cells, and the associated stromal cells in the tumor microenvironment (TME) [7–9]. More sophisticated 3D in vitro models (e.g., spheroids, organoids) have been developed to address the need for a 3D physiological structure. However, these models still miss some important features, such as the presence of a flow and mechanical cues, like the shear stress [10–12]. Conversely, in vivo animal models offer a better resource to overcome the limits of the 2D models. They allow the assessment of tumor growth and the response to drug treatments [13, 14]. Nevertheless, it is recognized that these models fail to recapitulate the specific human TME [15]. Flourishing literature highlighted the important role of the TME in supporting and influencing tumor behavior, thus becoming an essential component in deciphering the pathways related to tumor development and progression [16]. In this scenario, organ-on-chip (OoC) platforms are emerging as innovative and advanced 3D approaches. Indeed, they usually host multiple cell types in a more in vivo-like microenvironment [17–19]. In the last years, cancer-on-chip (CoC) platforms have been developed with the purpose to emulate the relevant physiological characteristics of the TME in vitro while controlling the mechanical stimulus, the flow, and the rate of chemical release at the cellular scale [20].

This review summarizes the important characteristics of CoC technology and application, focusing on the main impacting primary cancers and their usual metastatic sites.

## Insight into carcinogenesis, metastatic cascade, and the role of tumor microenvironment

### Carcinogenesis and the metastatic cascade

**Fig. 1:**
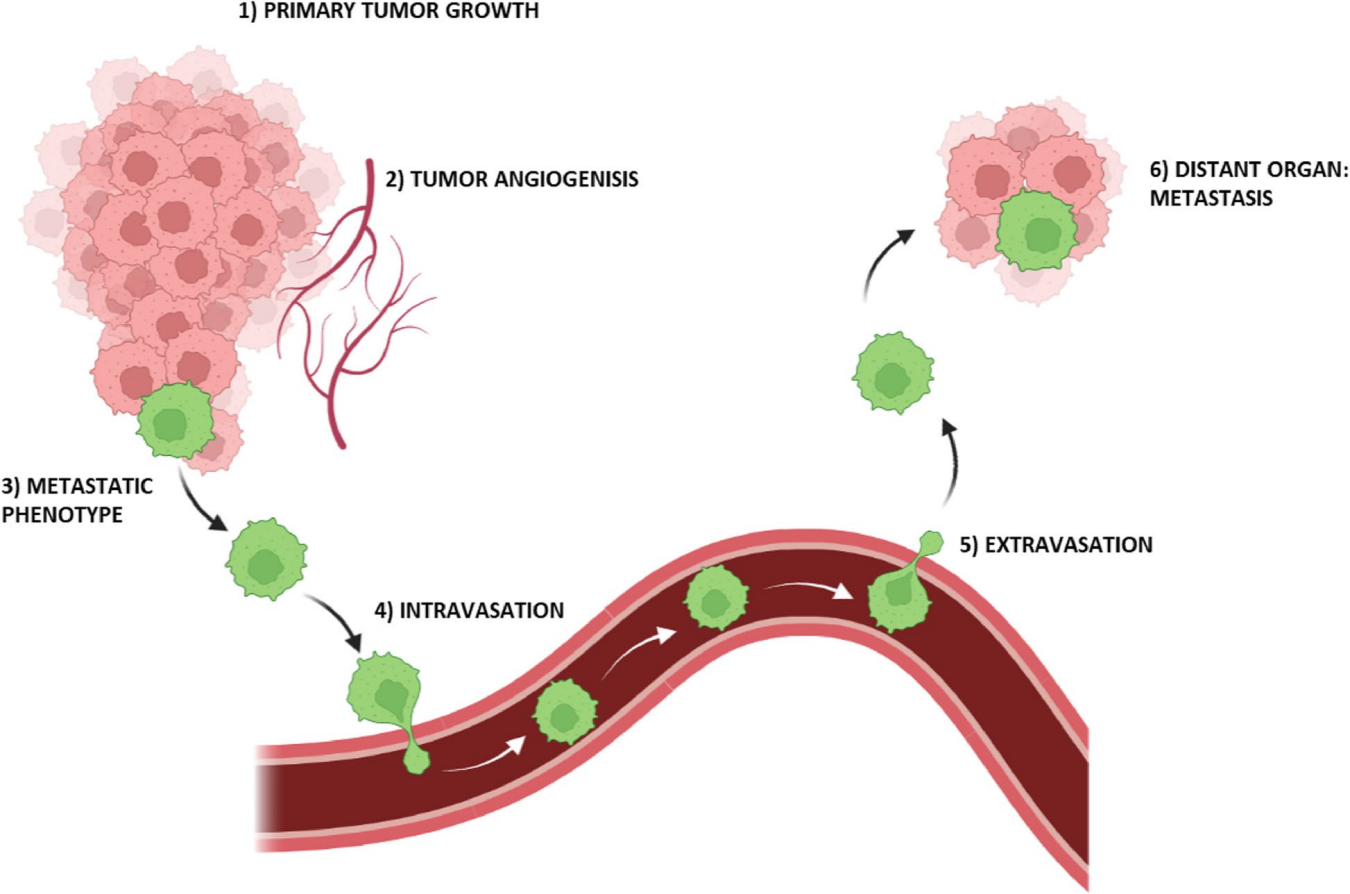
The carcinogenesis and the metastatic cascade. The carcinogenesis and the metastatic cascade are complex processes that comprise the mechanisms associated with the primary tumor and its colonization of other organs (metastasis). The first phase is the primary tumor growth (1) followed by the generation of new capillary blood vessels (2), a crucial step for tumor progression and invasion. Once the cancer cells undergo the epithelial-to-mesenchymal transition (EMT), they acquire the metastatic phenotype (3), which allows the cells to move and enter the blood vessels through intravasation (4) and leave them (extravasation, 5) when they reach distant organs, the metastatic sites (6)

Carcinogenesis is a complex process by which normal cells undergo genetic and epigenetic alterations leading to the development of cancer. These changes enable the cancer cells to evade regulatory mechanisms, invade surrounding tissues, and potentially spread to distant organs through metastasis [21]. The metastatic cascade is a complex and dynamic process involving a series of steps through which tumor cells disseminate from the primary tumor site to distant organs (Fig. 1) [22]. Intravasation is a critical step in the metastatic cascade, where cancer cells invade and cross endothelial barriers or lymphatic vessels to enter the circulatory system. This process involves changes in tumor cell adhesion molecules, cytoskeletal rearrangements, and the secretion of proteolytic enzymes that degrade the extracellular matrix. Subsequently, extravasation occurs, whereby tumor cells exit the bloodstream, adhere to the endothelial cells, migrate through the vessel walls, and establish secondary tumors in new tissue environments [23]. This highlights the importance of a deeper understanding of the molecular and cellular mechanisms underlying each stage of the metastatic cascade to develop effective therapeutic strategies.

### Tumor microenvironment

The TME is a complex and dynamic environment recognized to have a pivotal role in tumor initiation and progression [24]. Stromal cells (e.g., fibroblasts, endothelial cells, lymphatic vascular network, pericytes), immune cells from the adaptative and innate immunity (e.g., T and B lymphocytes, tumor-associated macrophages, natural killer cells), and extracellular matrix (ECM) components establish a bi-directional and complex cross-talk with tumor cells. This leads to the regulation of several cellular processes that promote tumor cell proliferation, invasion, and metastatization [16, 25].

Cancer-associated immune cells are recruited in the tumor niche in response to several chemokines and cytokines released from tumor cells, such as CCL2, IL-8, CXCL12, and CCL5. Consequently, an aberrant inflammatory response is triggered, and a strong immunosuppressive niche is induced [26, 27]. Indeed, the infiltration, density, or type of tumor-infiltrated immune cells is reported to have a prognostic value for several cancers [28–31].

Among the stromal components, cancer-associated fibroblasts (CAFs) represent one of the most predominant cell populations within the TME. They constitute a heterogeneous group of cells originating from different sources, such as tissue-resident fibroblasts, stellate cells, bone marrow mesenchymal stem cells, and pericytes [16, 32]. During tumorigenesis, fibroblasts are recruited to the tumor site due to the release of factors from neoplastic cells within the TME, such as fibroblast growth factors (FGF) and platelet-derived growth factors (PDGF) [33, 34]. Once CAFs are recruited and activated in the tumor site, an intensive mutual relationship is established between them and tumor cells. Extracellular components are secreted from CAFs modifying the surrounding TME and influencing the tumor cell behavior. CAFs release various metalloproteinases (e.g., MMP2, MMP9) influencing the remodeling of the ECM; growth factors, and cytokines are also let out such as CCL7, the transforming growth factor beta (TGFβ) and the stromal cell-derived factor 1 (SDF-1). These molecules promote tumor proliferation, spreading, and aggressiveness [35, 36]. CAFs are linked to high chemoresistance and a poor prognosis in many solid tumors [37–40].

Among the different cell types populating the TME, endothelial cells (ECs) also play a pivotal role in tumor progression and metastasis. Indeed, they respond to pro-angiogenic signals mainly released by tumor cells and in turn secrete several molecules that promote the sprouting of new blood vessels [41]. Through this process, namely angiogenesis, ECs provide oxygen and nutrients to the growing tumor, enabling its survival and expansion [42]. Furthermore, ECs actively regulate the recruitment and activation of the immune cells within the TME, while also contributing to tumor immunosuppression through the secretion of inflammatory cytokines, and angiogenic factors and exerting antigen-presenting functions [41, 43]. Notably, the tumor ECs have been shown to exhibit distinct molecular characteristics, including the upregulation of adhesion molecules and enhanced permeability, enhancing tumor cell adhesion on the ECs and dissemination to distant organs [44].

The ECM represents an essential component of the TME, which provides structural support to tumor cells, by creating a complex network of proteins (e.g., collagens, fibronectin) able to influence tumor growth and invasion [45]. Indeed, the different ECM components interact with the tumor cells, fostering pro-survival and pro-proliferation signals to cancer cells through the action of integrins, ECM transmembrane proteins, which act as mechanotransducers, thereby deeply influencing tumor cell behavior [46]. Moreover, the increased tissue stiffness, derived from a dense and abundant tumor ECM, promotes the development of a physical barrier that could hamper the diffusion of anti-cancer drugs and essential nutrients and oxygen, fostering the establishment of a hypoxic environment [45, 47]. These mechanisms further increase the malignant behavior of tumor cells, due to the activation of several pathways involved in tumor cell proliferation, plasticity, and invasion [45, 47].

In the last years, increasing attention has been focused on tumor extracellular vesicles (EVs), as critical mediators of cell-to-cell communication within the TME [48]. These small membrane-bound vesicles, released by both tumor cells and stromal cells, transport proteins, nucleic acids, lipids, and several signaling molecules to neighboring or distant cells, promoting cell proliferation, angiogenesis, immune escape, and metastasis [49]. Furthermore, EVs are employed by sensitive cells to transfer drug resistance to chemosensitive cells, by delivering multi-drug resistance proteins [50, 51].

In such a scenario, great efforts are focused on developing advanced culture models, such as organ-on-chips (specifically, cancer-on-chips). The main goal is to reproduce the key in vivo TME interactions, provide a deep understanding of the underlying molecular pathways, and identify new targeted therapeutic strategies [17, 52].

## Organ-on-chip technology: microengineering joins biology

### Definition and key characteristics

An organ-on-chip “is a fit-purpose fabricated micro-fluidic-based device, containing living engineered organ substructures in a controlled micro- or nanoenvironment, that recapitulate one or more aspects of the dynamics, functionalities and (patho)physiological response of an organ in vivo, in real-time monitoring mode” [53]. Indeed, these tiny devices host living cells in microchambers perfused thanks to the presence of hollow microchannels. The “chip” word derives from the adaptation of the photolithographic techniques used in the computer microchips, which allow features at micro and nanoscale. These small dimensions permit obtaining highly controlled environments that resemble those of human cells [54]. Three key features characterize this technology [55]:

1. the organization of the cells in an in vivo-like arrangement;
2. the choice to culture multiple cell types to better replicate the human conditions;
3. the implementation of biochemical and biophysical stimuli to resemble the organs’ or tissues’ functions.

### OoCs’ environment

The micro- and nanoenvironments are responsible for the precise tuning of the fluids inside the OoCs’ channels as well as for the spatiotemporal control of gradients (e.g., chemical or nutrients). These peculiar characteristics are one of the major contributions to the success of OoC technology. Indeed, OoCs allow replicating and controlling of similar-human physiological cues, such as perfusion (laminar, pulsatile, and interstitial flow), physical forces (compression and tension), fluid shear stress, cyclic strain, and biochemical gradients of specific compounds [56]. Laminar flow is present in small vessels of organs and tissues and has a pivotal role in reproducing their physiology and pathophysiology. In the microfluidic systems, luminal fluid shear stress is controlled allowing the study of their role in different biological peculiarities, such as the reorganization of the actin cytoskeleton [57], the translocation of proteins [58], and the modulation of angiogenesis [59]. Studies were also performed to assess how capillary laminar fluid flow can impact the metastatic cells in their cycle progression, motility, and phenotypic changes [60, 61]. Laminar flow is usually generated using gravity-driven devices, pressure regulators, and syringe pumps [62]. Pulsatile flow is applied to microfluidic systems that aim to reproduce the human vessels and the pulsatile blood flow [63, 64]. Reproducing the physiological nature of the human vascular system is important when the connection between organs is established [65] and when studying the properties of endothelial cells in normal or diseased conditions [66]. This type of flow is typically actuated thanks to peristaltic pumps (also embedded in the chips [67]), syringes, and pneumatic pumps [62]. Interstitial flow happens inside or around a 3D ECM and has important effects on different aspects of cells’ functions, like their motility [68]. This type of flow has a role in the cancer cells’ metastasis and how they access and shape other environments [69]. Hydrostatic pressure-driven flow is the most used to reproduce this kind of flow [62]. Compression is a physical cue indispensable for many tissues and organs, such as the skin and heart. Pressure devices have been implemented with microfluidic devices to reproduce the compressive stimuli and help in the formation of specific tissue and organ [70]. Vacuum and syringe pumps are also applied to reproduce the cyclic strain that specific organs experience in the human body [62]. Connective tissue and lungs are just an example where this stimulus is necessary [71]. Organ and tissue microarchitectures (physiological or not) are simulated in the organ-on-chips thanks to the use of ECM coatings or hydrogels in which cells are seeded to resemble the human biological 3D structures [72, 73]. Indeed, organ-on-chips integrate with or enhance other 3D models, such as those based on scaffolds (hydrogels, porous scaffolds) [74–76] and on cells (spheroids, organoids) [77, 78]. Table 1 provides an overview of the main characteristics of these 3D systems [79, 80]. ECM-like hydrogels work as a 3D cell culture framework when inside an OoC. They provide mechanical support thanks to their porosity, water retention, and stiffness [81]. Natural (e.g., collagen, fibrin) and synthetic (e.g., poly(ethylene glycol), polyacrylamide) hydrogels are both used in combination with OoCs [82, 83]. Natural hydrogels have different cell binding sites and growth factors that influence cellular behaviors. However, they usually show poor mechanical properties. On the other side, synthetic hydrogels' mechanical and chemical properties can be easily tuned, but they lack bioactive molecules to support specific cell functions [81]. Another scaffold technology used in the OoC application is that obtained with electrospun fibers. Specific electrospinning techniques have been developed to introduce the polymeric fibers at micro- or nanometric scale inside organ-on-chip models [84] to have adequate support for cell growth [85]. Spheroids and organoids are the most common cell-based options to obtain the cell-ECM interactions needed in the OoCs [79]. Spheroids are the simplest 3D culture method coming from the spontaneous aggregation of differentiated cells, while organoids have a specific development process starting from either embryonic stem cells, induced pluripotent stem cells, or adult stem cells [79, 80]. The replica of the physiological micro- and nanoenvironments comprises also the tissue interfaces and endothelialized vascular channels [86, 87]. The implementation of these last biological features in the OoCs can also be achieved through the implementation of the 3D structures described above [88, 89].

**Table 1:** General description of 3D scaffold- and cell-based models [79, 80] that can be combined with organ-on-chips

3D Model	Description	Pros	Cons
Spheroids	Multiple cells are forced to form an aggregate thanks to cell–cell attachment-driven force	Simple High throughput Co-culture of different cells Scalable	A limited number of cell types No control over cells arrangement Missing perfusion
Organoids	3D structure derived from pluripotent stem cells, adult stem cells, and somatic cells that self-organize to mimic the structural and basic function of organs and tissues	Mimic the development stages of a specific organ/tissue Co-culture of different cells Relevant physiological structure and function	Missing standardization protocols Long development process No control over cells arrangement Missing perfusion
Scaffolds/Hydrogel	Natural or synthetic polymers that constitute a matrix or support for cell culture	Simple Standardized protocols Gradients of nutrients and chemical substances Co-culture of different cells	Specific molds can be required for scaffold/hydrogel construction Not full replication of in vivo ECM

### OoCs’ technology

**Fig. 2:**
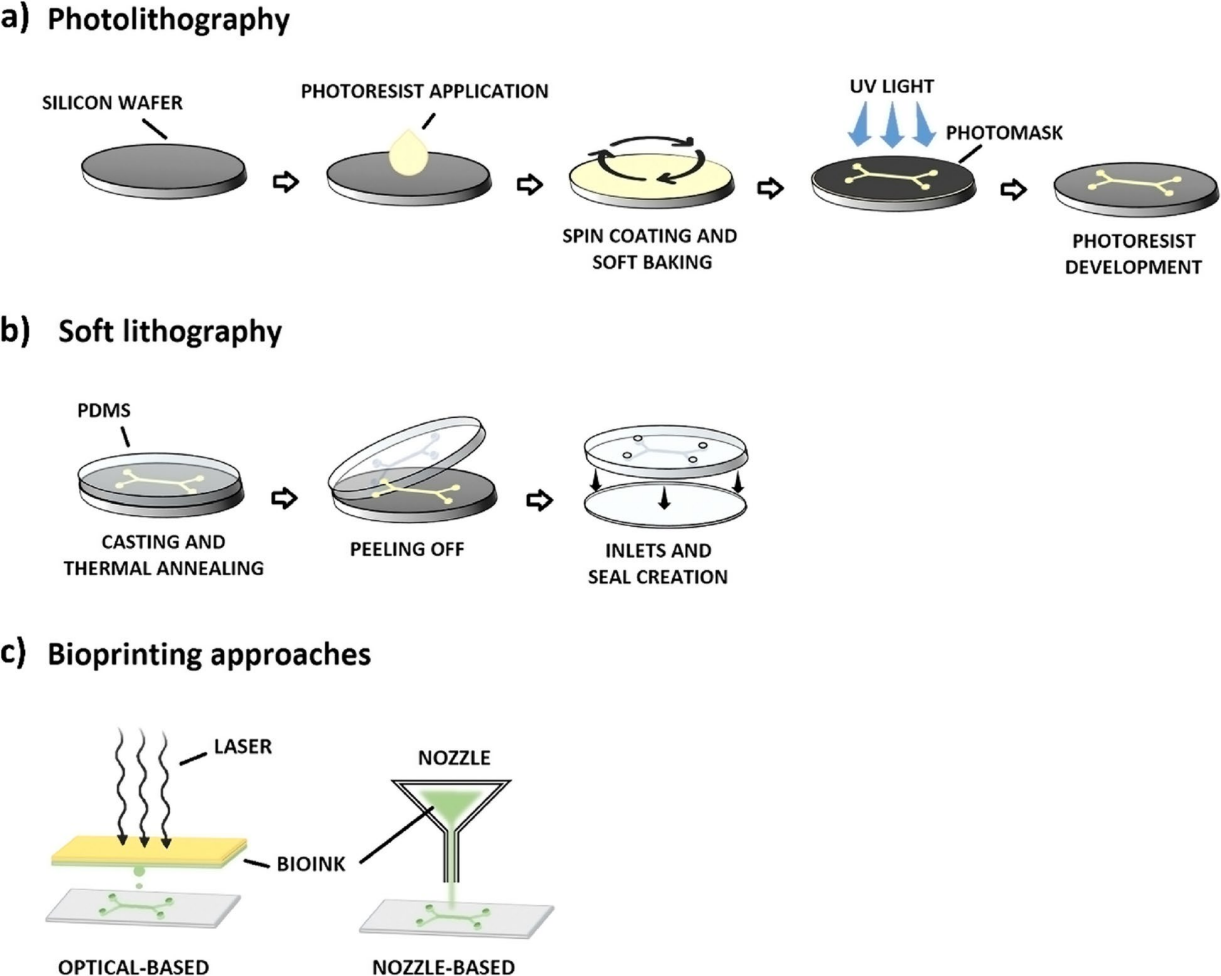
The two main microfabrication techniques used to generate organs-on-chip. **a** Photolithography is the core microfabrication technique used to transfer micro- and nanoscale patterns to photosensitive materials by optical radiation. A silicon wafer is used as support for the photo-sensitive material, which is generally called photoresist. After its application on the wafer’s surface, the wafer is spin-coated to obtain a thin uniform film of the photoresist, which is then brought in contact with a photomask that reproduces the desired pattern. The photoresist crosslink in the parts exposed to high-intensity ultraviolet (UV) light; while the covered photoresist is removed by a chemical agent. The negative design of the mask is now reproduced on the silicon master. **b** Soft lithography allows the fabrication of elastomeric molds using a replica molding technique. The PDMS is cast against the bas-relief pattern of the silicon master photoresist. After a thermal phase, the resulting substrate is peeled off showing the 3D pattern of the original master. The microfluidic device is then generated by creating the needed features, e.g., the inlets, and by bonding it to a PDMS or glass slab. **c** 3D bioprinting constructs microfluidic devices using a fast and automated process. In the bioprinting nozzle-based approach, the bioink is extruded through a nozzle moved by a computer-controlled arm to create 3D shapes. Superior resolutions are obtained using optical-based approaches where laser exposure solidifies the bioink through a crosslinking reaction

The production of these microsystems mainly relies on silicon rubber polydimethylsiloxane (PDMS), an easy-to-use material that paved the way for the application of OoC technology by many research groups. PDMS characteristics, such as high gas permeability, optical transparency, and high flexibility, make it suitable for cell culture. Microfabrication techniques (photolithography, soft lithography, and replica molding) are easily applied to PDMS to generate patterns and structures relevant to physiological conditions (Fig. 2A and B) [54, 56]. Indeed, OoCs’ features shapes and sizes (e.g., hollow channels) on the scale of nm or μm are obtained thanks to photo- and soft lithography. Such dimensions are in the same range as those sensed by the living cells in the human body [56]. Other techniques are also employed to produce OoCs with the use of different materials. Bioprinting is one of the most promising fabrication approaches, where a cell-laden bioink is printed with supporting materials to construct functional tissues and organs [90, 91]. Different bioprinting methods are available, from the simplest nozzle-based approaches to the most sophisticated optical-based techniques (Fig. 2C) [92, 93]. Notwithstanding the production methods, OoCs can be more biologically functional thanks to the introduction of micro- and biosensors. Some examples are the transepithelial/endothelial resistance (TEER) sensors for analyzing barrier model integrity [94], multielectrode arrays to monitor neuronal networks and cardiac tissues [95, 96], and optochemical sensors to study cell metabolism [97]. Various OoCs were produced in the last years, starting from those replicating the key physiological functional units of whole human organs or tissues, such as the lung [98, 99], liver [100, 101], gut [102, 103], heart [104, 105], skin [106, 107], kidney [108], muscle [109, 110] and the blood–brain barrier [111, 112]. OoCs mimicked also diseased and pathologic conditions like acute SARS-CoV-2 [113, 114], asthma [115], ischemia [116, 117], cardiac fibrosis [118, 119], inflammatory bowel disease (IBD) [120], fatty liver disease (FLD) [121, 122], diabetes [123, 124], Alzheimer’s disease [125] and CoC platforms, recapitulating specific primary tumors with their metastatic site.

## Cancer-on-chip to study primary tumors and the associated metastatic sites

In recent years, there has been a significant advancement in the development of various models of cancer-on-chip (CoC) systems, which aim to closely resemble the primary and metastatic tumor microenvironment (TME) in an in vivo-like manner. These models have primarily focused on studying specific stages of carcinogenesis, ranging from tumor growth to the metastatic process. Among these models, microfluidic systems have been demonstrated to be able to mimic the in vivo tumor conditions than traditional 2D systems [126, 127].

### CoC and the primary tumor

The role of extracellular vesicles (EVs) in influencing primary tumor behavior and growth is of great interest. To investigate their release within a solid tumor model, researchers have developed an intriguing organ-on-chip platform called EV microbioreactors (EVμBRs) [128]. The EVμBRs have demonstrated the ability to replicate cellular physiology and heterogeneity, providing a valuable in vitro tool for investigating the implications of EVs in tumor behavior. Tumor angiogenesis, the formation of new blood vessels in the TME, is another important process in carcinogenesis. Microfluidic devices have been employed to gain deeper insights into this complex mechanism, enabling the study of early stages of tumor growth and the development of the tumor microvascular network [129, 130]. Noteworthy, the spread of tumor cells is closely associated with a phenomenon called epithelial-to-mesenchymal transition (EMT), in which epithelial cells undergo changes and acquire a more mesenchymal phenotype. In one study, researchers generated an EMT index using tumor-derived EVs isolated in a microfluidic chip to evaluate metastatic risk [131]. Other factors involved in promoting EMT have been investigated using organ-on-chip systems, including the influence of mechanical stimuli such as flow-induced hydrodynamic shear stress and the role of hypoxia, in more reliable in vivo conditions [132–134].

### CoC and the metastatic process

Recently, increasing interest has been in deciphering the mechanisms underlying the metastatic process and investigating the specific organs where tumor cells prefer to metastasize. Several multi-organ microfluidic chips were developed to investigate metastatic events. In a pilot study using a metastasis-on-a-chip, it was observed that primary colorectal cancer cells tend to preferentially metastasize to the lung and liver constructs when fluidically linked to them, consistent with observations in humans [135]. Similar results were obtained when studying lung cancer metastasis using a multi-organs-on-a-chip, considering the liver, lung, and brain as potential metastatic sites [136]. After the settlement of metastatic cells in these organs, specific factors associated with cellular damage were released. These findings align with the “seed and soil” theory, which suggests that tumor cells (the “seed”) can colonize and establish secondary tumors in specific organs or tissues (the “soil”) [137]. The preference of tumor cells for specific organs or tissues is influenced by the molecular characteristics of the cells and the microenvironmental factors of the target site, such as extracellular matrix composition, immune cell presence, and molecular signaling molecules [138].

### CoC for the most common cancers

The aforementioned examples highlight the ability of these innovative microfluidic devices to recapitulate crucial aspects of different tumor processes, including development, growth, and metastasis and their applicability to better elucidate these processes. A deeper understanding of tumor cell characteristics and the microenvironments within CoCs holds the potential to provide valuable insights into the factors that either promote or hinder metastatic spread. This knowledge can contribute to the development of more effective strategies for the treatment of metastatic diseases.

A comprehensive review was undertaken to examine CoC models developed for the most common cancers worldwide, such as breast, lung, pancreas, colon, and liver [139, 140]. This review critically discusses the strengths and limitations of these CoC platforms, while also exploring their potential future applications in advancing our understanding of tumor mechanisms.

## Breast cancer

### Framework

In 2020, 2.3 million women were diagnosed with breast cancer and more than half a million died globally [141]. Breast cancer develops both in the duct and in the lobule of the glandular tissue of the breast. The treatment of primary breast cancer can be highly effective, but there are difficulties with the metastasis sites [141]. Indeed, breast cancer tends to metastasize in the brain, lungs, bones, and liver, leading to the death of many patients [142]. There are several breast cancer subtypes among which triple-negative breast cancer is the most challenging to treat. Intensive efforts are focused on developing new treatments for this tumor type as well as patient-specific therapeutic applications.

### CoCs to study the breast primary tumor

**Fig. 3:**
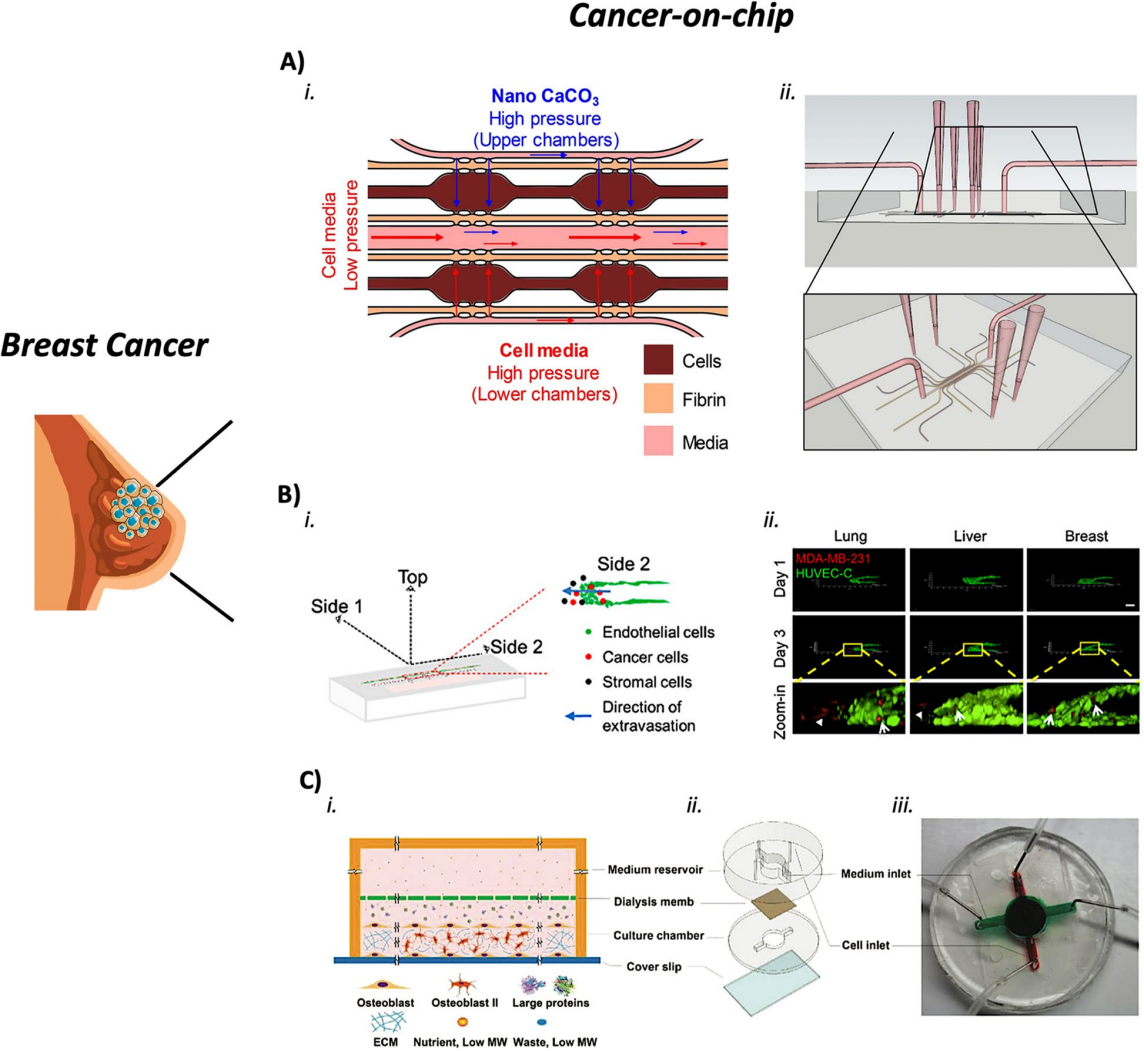
Examples of breast cancer-on-chips. **a** Bifurcated chip to study a possible solution for the acidification of the primary tumor environment. (i) Design of the microfluidic device: the upper chambers are loaded with CaCO_3_ nanoparticles able to neutralize the acidification of the TME. (ii) Chip setup. Pipette tips are used to feed the upper and lower chambers, while the middle channel (which ensures the separation between the control and experimental compartments) is connected to a syringe pump. Adapted from [144] with permissions from Scientific Reports. **b** Organ-on-chip model to analyze the tissue-specific breast cancer extravasation. (i) Schematic illustration of the extravasation chip with the Side 2 view highlighted. (ii) Z-stack projection images of Side 2 view showing HUVEC-C endothelial monolayers (green), extravasated (arrowhead), and associated (arrow) human breast cancer cells (MDA-MB-231, red) into the lung, liver, or breast microenvironments. Adapted from [145] with permissions from Biotechnology & Bioengineering. **c** Miniaturized bone-on-a-chip to study breast cancer bone metastasis. (i) Schematic of the simultaneous-growth-and-dialysis mechanism. Low-molecular-weight nutrients and metabolic waste move continuously through the dialysis membrane. While large bone matrix-building proteins accumulate in the bottom chamber contributing to the spontaneous formation of the osteoblastic tissue. (ii) Exploded view of the bone-on-a-chip. (iii) Injected inks highlight the central circular area of the assembled chip. Dialysis occurs in this space. Adapted from [146] with permissions from Small

In a study, triple-negative breast cancer cell lines were cultured in an organ-on-chip platform based on the standard 384-well plate. The goal was to test breast cancer therapies [143]. Characterization in terms of seeding densities, ECM composition, and biochemical conditions was performed for three distinct breast-cancer cell lines. These cells were exposed to a series of anticancer agents (paclitaxel, olaparib, and cisplatin) and compared to 2D models treated with the same drugs. The executed tests showed a different behavior between 2D and organ-on-chip models. Especially, the response to cisplatin was more like the physiological one in the cell lines treated inside the organ-on-chips. This result was confirmed using primary tumor cells. Therefore, this technology could be a promising tool for personalized medicine and/or could help in the selection of suitable therapies. A lot was done to understand the mechanisms of tumor cell invasiveness and aggressiveness. For example, acidification of the primary tumor environment is considered one of the causes that induce an invasive behavior in cells [144]. The TME acidification and its possible neutralization using CaCO_3_ nanoparticles (nanoCaCO_3_) were the main core of a study using a bifurcated chip, with the experimental and control conditions on the same cancer-on-chip [144]. Moreover, hydrostatic pressure was highly controlled so to generate physiological flow inside the channels. Indeed, it was shown that tumor growth and migration were inhibited thanks to a constant buffering of nanoCaCO_3_ (Fig. 3A). NanoCaCO_3_ particles induced tumor cell reprogramming by altering the TME pH. Coupling this methodology with drugs that are effective in an acid environment can open the door to new therapeutic strategies.

### Breast CoCs for the metastatic behavior

Important aspects of breast cancer metastasis are intravasation and extravasation events. Intravasation refers to tumor cells entering the blood circulation, while extravasation refers to tumor cells exiting from the bloodstream and creating a new colony. The interactions between metastatic tumor cells and the blood vasculature were studied in an engineered 3D vasculature. This structure was generated through rapid multilayer microfabrication, where subpopulations of triple-negative breast cancer cells were seeded surrounded by osteoblasts, bone marrow-derived mesenchymal stem cells, or lung fibroblasts [147]. Experiments were performed mimicking the lung or the bone microenvironment. The obtained results highlighted that the osteoblasts play a crucial role in the selective extravasation of bone MDA-231, a specific breast tumor subpopulation. Therefore, this technology proved to be useful in the investigation of organotrophic metastasis and a helpful tool in identifying targets and treatment strategies to benefit patients. Another study was performed by developing two novel cancer-on-chips to analyze the tissue-specific breast cancer invasion/chemotaxis and extravasation [145]. Liver, lung, and breast microenvironments were mimicked to distinguish invasion/chemotaxis toward these different tissues. It was observed that metastatic breast cancer cells tend to invade the lung and liver more than the breast tissue. Moreover, the lung-specific metastatic cell subpopulation had a higher invasion behavior in the lung microenvironment than other metastatic cell subpopulations, like the bone one. Finally, an extravasation model was implemented, which comprised also an intact endothelial monolayer. It was shown that metastatic breast cancer cells are prone to cross the endothelial barrier when the lung microenvironment is simulated (Fig. 3B). These results could help to improve cancer diagnosis and select the best therapeutic option. Other factors can play an important role in breast cancer metastasis. For example, a study analyzed the role of the sympathetic nervous system (SNS) in the modulation of breast cancer metastasis [148]. In this research, a human metastasis-on-chip platform was developed to reproduce the effect of sympathetic activation on the dynamic crosstalk between bone tropic breast cancer cells and osteoclasts. It was shown that bone tropic breast cancer cells received synergistic inputs from neurons and osteoclasts. The osteoclasts increased pro-inflammatory cytokines that are important for the progression of breast cancer bone metastasis and osteoclastogenesis. This finding proved the importance of correctly reproducing the interactions of the specific metastatic microenvironment. Moreover, this microfluidic model allowed stopping communications among the three different compartments, bone tropic breast cancer cells, sympathetic neurons, and osteoclasts. In particular, the above-described effects on breast cancer bone metastasis and osteoclasts were not present when the interaction between bone and neuron compartment was interrupted, even if the levels of the studied pro-inflammatory cytokines remained quite stable. Bone metastasis generated by metastatic breast cancer cells was also studied using a miniaturized bone-on-a-chip (Fig. 3C) [146]. A naturally thick mineralized 3D tissue was generated by applying the principle of simultaneous growth dialysis. The resulting bone tissue provided the necessary microenvironment for the colonization of metastatic breast cancer cells. Both metastatic and metastasis-suppressed breast cancer cells were introduced into the developed osteoblastic tissue. The metastasis-suppressed breast cancer cells showed the expected dormant behavior with limited metastases. On the other hand, the metastatic cells replicated key features and characteristics usually observed in vivo, such as the invasion of the mineralized tissue apical layer, invadopodia protrusion, and formation of the so-called “Indian files” formed by the invading cancer cells. Therefore, the developed bone-on-a-chip proved to be a physiologically relevant model for the study of breast cancer bone metastasis in vitro.

### Breast CoCs’ advantages and limitations

In summary, the described breast cancer models have advantages in terms of more suitable therapies’ selection in the context of personalized medicine [143], in the study of the mechanisms behind tumor cells’ invasive behavior [144], in the analysis of the intravasation and extravasation events [145, 147], and in the replication of the metastatic microenvironment to identify the key phenomena [146, 148]. Organotropism, the non-random process where distant metastases are distributed to specific organs, can be elucidated thanks to the use of CoCs giving hints to identify better therapies for patients [138, 145]. However, these studies were not always able to replicate the 3D structure of the tumor microenvironment [143] and the associated stromal cells that are pivotal for the correct representation of the TME [144].

## Lung cancer

### Framework

Lung cancer is one of the most diagnosed cancers worldwide with a high mortality rate [149]. Public health measures have been implemented in industrialized countries to reduce smoking, which is the main cause of such cancer. However, a high smoking incidence is still present in low-income nations. On the other hand, the lung cancer subtype adenocarcinoma continues to occur in people that have never smoked [149]. Lung cancer often metastasizes in the liver, brain, bones, breast, and kidney, with a very low survival rate for patients with metastasis. Therefore, the high and aggressive progression and the well-known resistance to chemotherapy have led to a search for better methods to investigate the mechanisms of lung cancer development and metastasis [150]. In this context, organ-on-chips represent a promising option to elucidate the underlying processes of lung cancer.

### CoCs to study the lung primary tumor

**Fig. 4:**
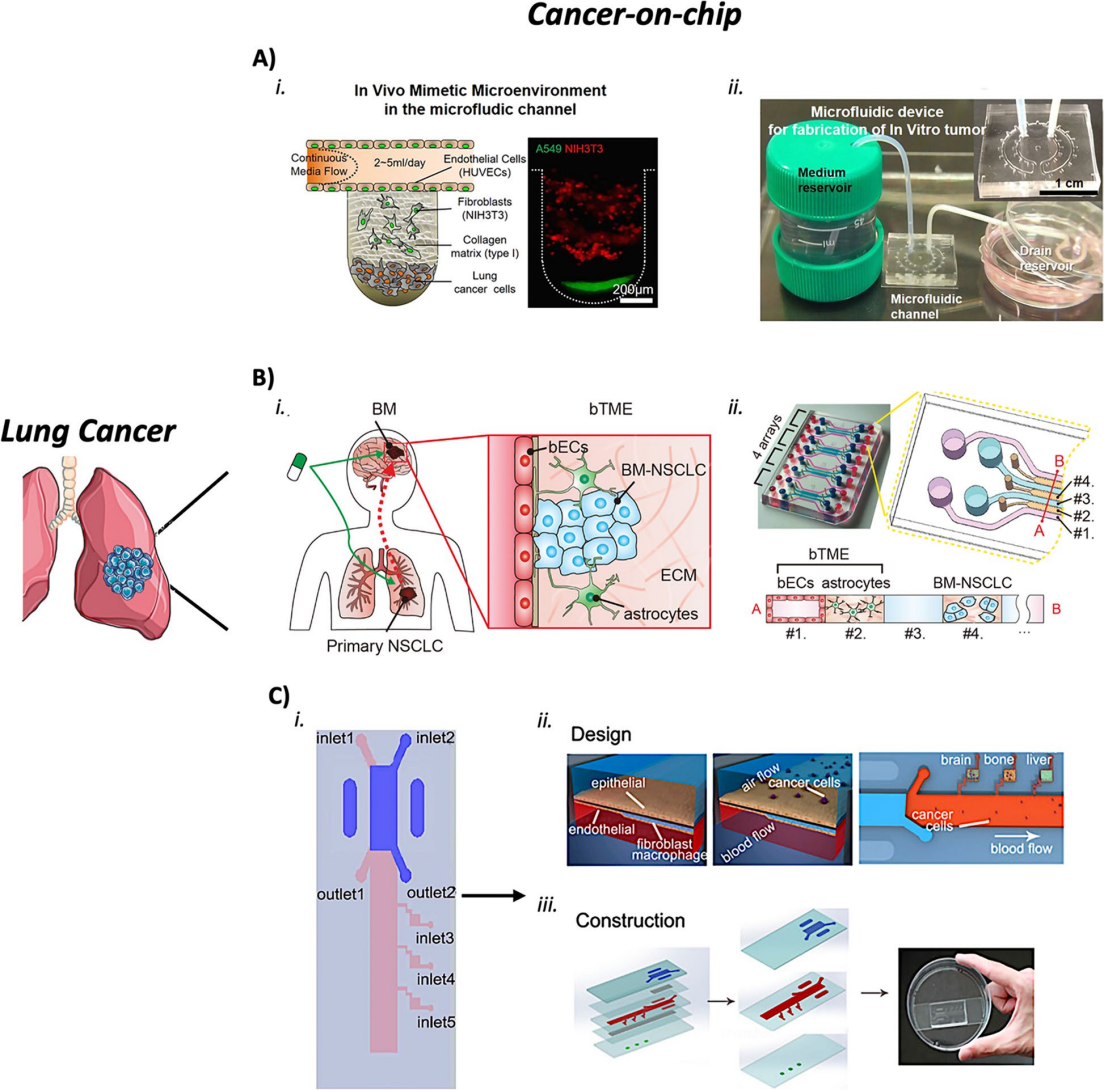
Examples of lung cancer-on-chips. **a** Microfluidic chip for the study of the role of the stromal cells in tumorigenesis. (i) An in vivo-simulating representation of the TME was achieved by integrating into the same microfluidic device stromal cells, fibroblasts, and endothelial cells surrounded by a 3D collagen matrix with a channel for the continuous flow of the culture medium. (ii) Overview of the main components interacting with the microfluidic device. Adapted from [153] with permissions from Scientific Reports. **b** Microfluidic device to recapitulate the metastatic brain niche. (i) Representation of the bTME composed of BM-NSCLC, cerebral microvascular endothelial cells, and primary human brain astrocytes. (ii) Configuration of the seven-channel microfluidic device with its cross-section showing where each cell type is cultured. Adapted from [154] with permissions from Advanced Science. **c** Multi-organs-on-a-chip for the study of different metastatic sites. (i) Schematic illustration of the multi-organs-on-a-chip comprising the primary site of cancer (the lung, in purple) and the three distant organs (inlet 3, inlet 4, and inlet 5). (ii) Representation of the chip lung structure, where a membrane divides the air compartment from the blood one. Lung cancer cells are co-cultured with human bronchial epithelial cells on the upper side of the membrane, while stromal cells (microvascular endothelial cells, fibroblasts, and macrophages) are seeded on the lower side. Metastatic lung cancer cells move along the blood channel to reach distant organs, the brain, bone, and the liver. (iii) Overview of the chip structure composed of three main layers and two microporous membranes. Adapted with permission from [136]. Copyright 2016 American Chemical Society

A study was performed to reproduce human orthotopic models of non-small cell lung cancer (NSCLC) in vitro [151]. The aim was to recapitulate the in vivo-like TME and investigate tumor growth using a lung-on-chip device made of two parallel channels separated by a porous membrane. Epithelial cells and a low density of NSCLC cells were cultured in one channel on the porous membrane, while human lung microvascular endothelial cells were cultured on all four walls of the facing channel, forming a hollow vascular lumen. A mechanical suction was applied to mimic normal breathing. Indeed, it was found that this mechanism significantly suppressed lung cancer growth. Resistance to tyrosine kinase inhibitor (TKI) therapy of patients showing specific mutations was successfully reproduced in this orthotopic device. This mechanism was never observed in the conventional 2D in vitro models. The simulation of the alveolar microenvironment to study lung cancer was achieved by implementing a poly(lactic-co-glycolic acid) (PLGA) electrospinning nanofiber membrane as the cell scaffold of a lung-on-chip [152]. A human NSCLC (A549) and a human fetal lung fibroblast (HFL1) cell lines were co-cultured in the chip device on the upper and lower sides of the membrane, respectively. This study evaluated the effect of gefitinib, a selective inhibitor of the epidermal growth factor receptor (EGFR)-tyrosine kinase. Significant resistance to drug treatment was observed in A549 and HFL1 cell co-culture, thus confirming the role of HFL1 cells in decreasing tumor cells’ sensitivity to chemotherapy. Moreover, A549 and HFL1 cells were co-cultured with endothelial cells (HUVEC). This experiment showed how A549 cells became strongly invasive destroying the endothelial barrier and starting the metastatic invasion process. Therefore, reproducing the TME is pivotal to obtaining a pathophysiological model. A 3D microfluidic lung cancer model was developed to investigate the role of the stromal cells in lung tumorigenesis and to resemble as much as possible the in vivo TME [153]. This in vitro lung cancer platform was established by tri-culturing endothelial cells, fibroblasts, and lung cancer cells within a 3D collagen matrix (Fig. 4A). The presence of fibroblasts allowed the formation of the tumor environment by regulating the biophysical and biochemical properties of the TME. Moreover, the vasculogenesis induced by fibroblasts was confirmed thanks to the establishment of a stable in vitro tumor model with a complex structure.

### Lung CoCs for the metastatic behavior

A lot was done to elucidate the mechanisms of the lung cancer metastasis process and to investigate the characteristics of the metastatic sites. In a seven-channel 3D microfluidic platform, brain metastatic non-small cell lung carcinoma (BM-NSCLC), cerebral microvascular endothelial cells, and primary human brain astrocytes were cultured together to reconstitute the brain tumor microenvironment (bTME) (Fig. 4B) [154]. The enhanced cancer cells’ survival was studied revealing the activation of specific pathways against the anti-cancer drugs. Moreover, cytokine-related intracellular pathways were discovered to be responsible for the acquired drug resistivity. This model elucidated the communication mechanisms among the different components of the bTME. A multi-organs-on-a-chip was developed including the primary site of cancer (the lung) and three different metastatic organs (the brain, bone, and liver) (Fig. 4C) [136]. Changes in lung cancer cells and expression of specific epithelial and stromal markers were identified as clear signals of tumor growth and cell invasive capacity. Cell–cell interactions during metastasis have been elucidated thanks to this microfluidic device.

### Lung CoCs’ advantages and limitations

In conclusion, the described lung cancer-on-chip models highlight the potential of these systems in studying tumor growth and its response to specific therapies [151, 152]. They confirm the important role of TME reproduction to resemble as much as possible the in vivo conditions [154] and describe the communication mechanisms and interactions among the different TME components in the metastatic organs [136, 154]. Better pathophysiological models could be obtained by introducing specific TME components that play a role in tumor progression [151, 152] and cancer-associated immune cells [154].

## Pancreatic cancer

### Framework

There are two main types of pancreatic cancer: exocrine and rare endocrine cancer. Exocrine Pancreatic Ductal Adenocarcinoma (PDAC) has one of the lowest survival rates [155]. Failure of current chemotherapeutics is reasonably due to the high molecular heterogeneity of PDAC and its intricate tumor microenvironment [156, 157]. A specific system is pivotal for drug discovery and personalized medicine in such cancer.

### CoCs to study the pancreatic primary tumor

**Fig. 5:**
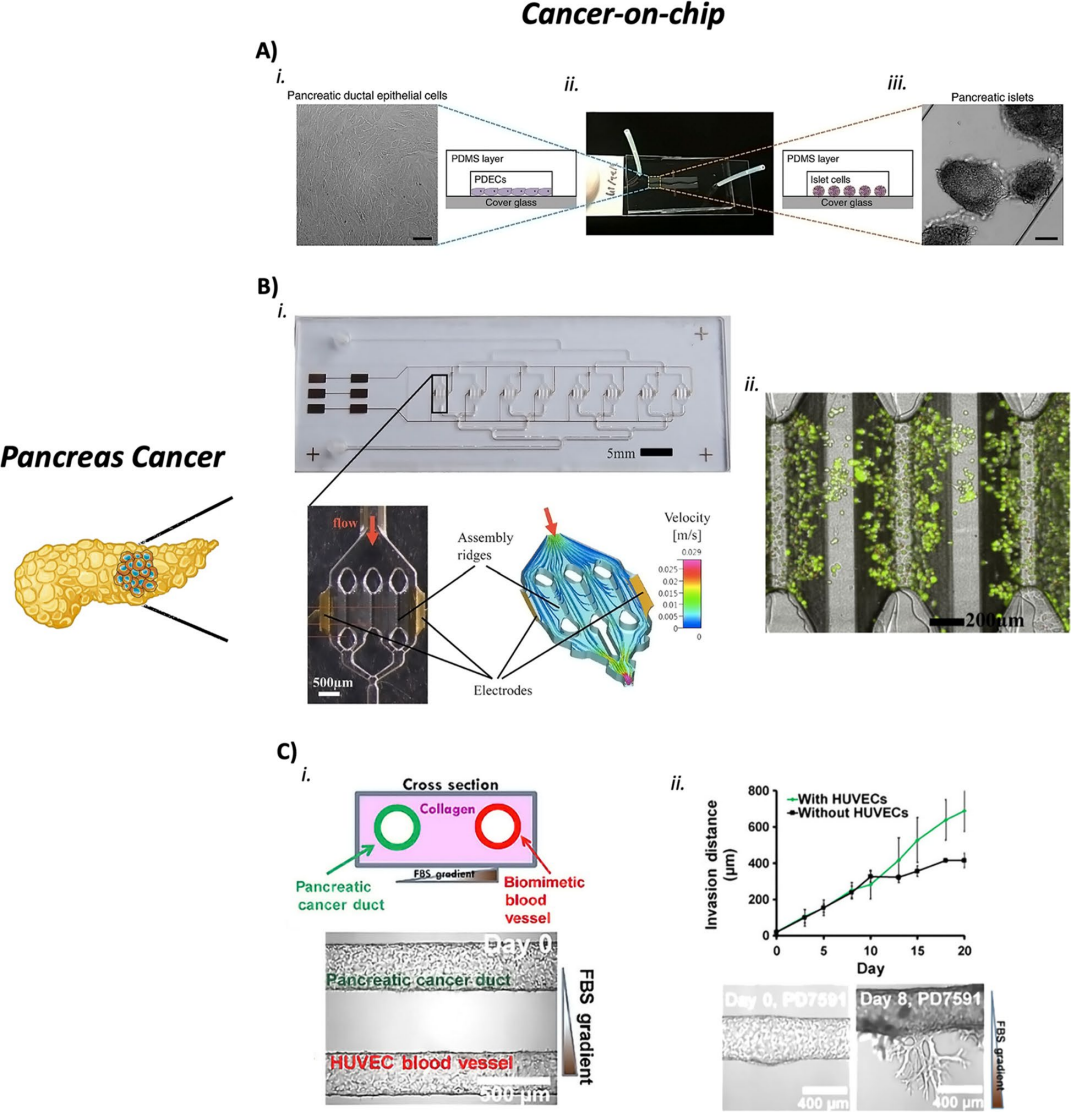
Examples of pancreatic cancer-on-chips. **a** Pancreas-on-a-chip to model fibrosis-related disorders. (i) Pancreatic ductal epithelial cells (PDCEs) were cultured inside the single-channel chip (ii) together with pancreatic islets (iii) to monitor the Cystic Fibrosis Transmembrane Conductance Regulator (CFTR) function. Adapted from [158] with permissions from Nature Communications. **b** HepaChip® for the diagnosis and prognosis of PDAC. (i) Image of the chip with the 8 culture chambers. The electrodes and ridges present in each chamber are shown together with flow velocity and trajectory simulation. (ii) Live/Dead of the PDAC cells after 146 h of culture inside the HepaChip®. Adapted from [159] with permissions from Scientific Reports. **c** Organ-on-chip to model the invasion of PDAC tumor cells to blood vessels. (i) Two hollow cylindrical channels in the microfluidic device mimic the blood vessel and the pancreatic cancer duct, respectively. Endothelial cells (HUVEC) were seeded in the perfusable vessel, while pancreatic cells were cultured in the cancer duct. (ii) Representation of the average invasion distance of the PDAC cell line PD7591 when an FBS gradient is established and with/without the HUVEC cells. Speed migration is increased when the HUVECs are present. Adapted from [161] with permissions from Science Advances

Few successful attempts have been published so far, notwithstanding a pancreas-on-a-chip was generated for the first time in 2015 [158]. This model was inflated with isolated patient-derived pancreatic ductal organoids mimicking pancreatic cell function and interface in situ (Fig. 5A). Even if this model didn´t recapitulate the pancreatic TME, it represents the first physiologic-like model of the pancreas. A study was carried out to develop a platform useful in the diagnosis and prognosis of PDAC, the so-called HepaChip® [159]. The obtained results demonstrate the feasibility of PDAC cell cultures in a microfluidic chamber under continuous and controlled perfusion (Fig. 5B). Moreover, the chemotherapeutic drug cisplatin was tested in the organ-on-chip model giving consistent responses with what is observed in vivo. Pancreatic patient-derived organoids were cultured in a microfluid scaffold platform called InVADE. This study aimed to elucidate the evolution of the PDAC stroma and its effects on drug bioavailability. The resulting vascularized human PDAC model also captured the hallmarks of an evolving TME thanks to a co-culture with human fibroblasts [160].

### Pancreatic CoCs for the metastatic behavior

The most frequent pancreatic cancer metastasis occurs in the liver, peritoneum, and lung (80%, 48%, and 45%, respectively) [162]. Indeed, pancreatic cancer expresses and secretes a plethora of proangiogenic factors [163], even if it is poorly vascularized [164]. In 2019, a 3D organotypic model helped to shed light on this mechanism (Fig. 5C). This model was able to recapitulate the invasion of PDAC tumor cells to blood vessels, showing how these cells can rapidly penetrate the lumen of blood vessels and ablate the endothelial cells [161].

### Pancreatic CoCs’ advantages and limitations

The reported examples show how these models can reproduce the main physiological features of the pancreas in vitro [158], the major characteristics of the PDAC TME, and its response to drug therapy [159, 160]. Moreover, pancreas cancer-on-chip models help better understand this cancer type's metastasis mechanisms [161]. However, still, too few studies have been published to better investigate this type of cancer and the related metastasis mechanisms, and the described ones lack important TME components and cancer-associated stromal cells [159].

## Colorectal cancer

### Framework

Colorectal cancer (CRC) represents the second leading cause of cancer-related death and the third most diagnosed cancer worldwide [165]. CRC is largely asymptomatic until it progresses to advanced stages characterized by distant metastasis and poor overall survival [166, 167]. The liver represents the most common CRC metastatic site: 25–30% of patients present colorectal liver metastasis (CLM) at the time of diagnosis or develop it after the primary tumor resection [168, 169]. Furthermore, most of these patients are not eligible for curative surgery at the time of diagnosis and they usually have a 5-year survival rate (< 15%) [170, 171]. This highlights the urgent need to improve drug treatments along with a deeper understanding of the biological mechanisms of CRC. In such a scenario, several OoCs were developed in the last years aimed to elucidate in depth the CRC molecular pathways, perform drug testing and understand the metastatic steps of this disease [135, 172–175].

### CoCs to study the colorectal primary tumor

**Fig. 6:**
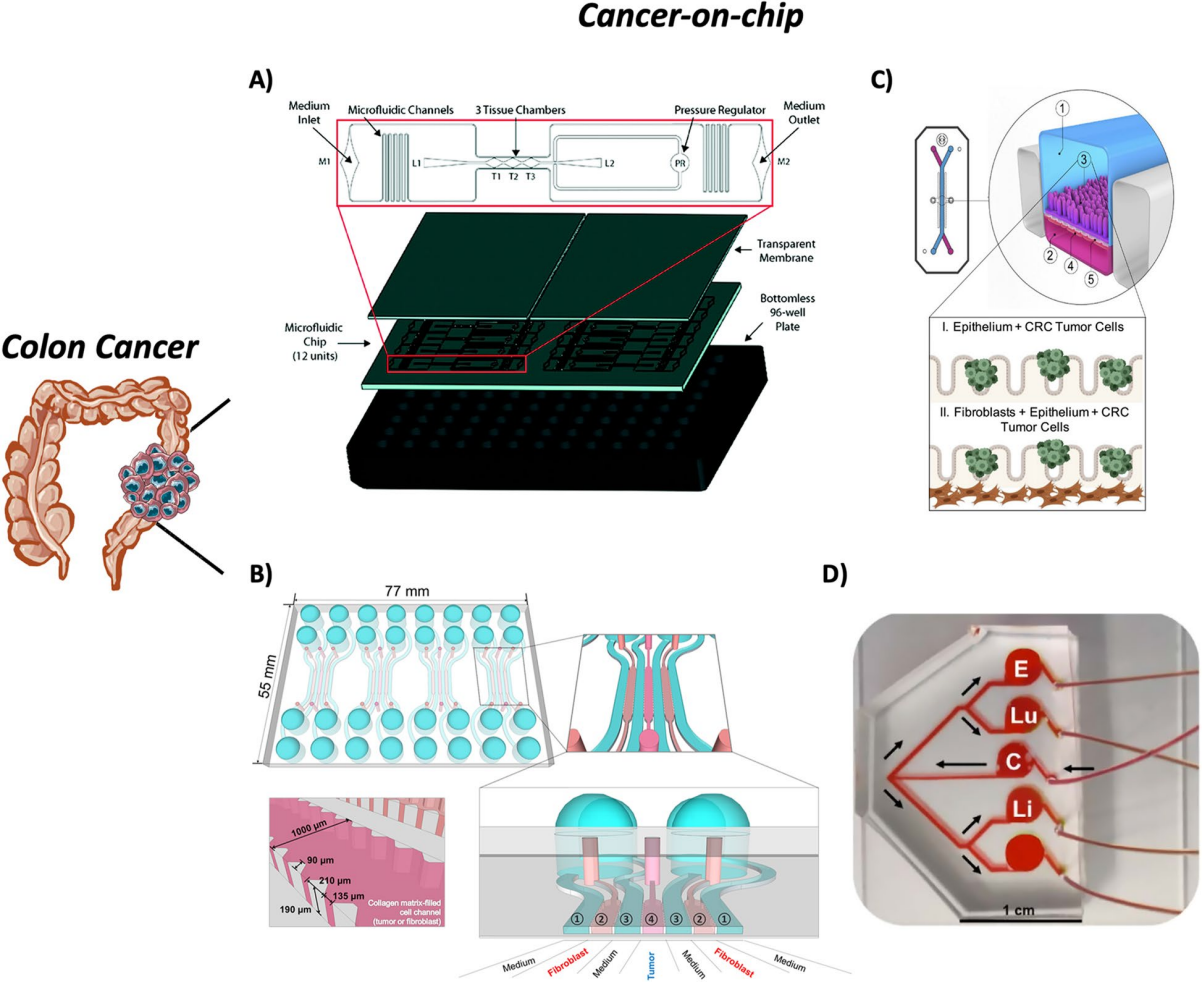
Examples of colon cancer-on-chips. **a** The vascularized micro-tumors (VMTs) are composed of 3 tissue chambers (T1-3), hosting CRC cells, fibroblasts, and endothelial cells. There is also a pressure regulator (PR) to prevent the gel rupture, two loading ports (L1-2), and two medium inlets and outlets (M1-2). The entire structure is bonded onto a bottomless 96-well plate. Reproduced from [172] with permission from The Royal Society of Chemistry. **b** Schematic representation of the microfluidic device for tumor and fibroblast cells co-culture composed of seven channels: three to host cells and four for the media. Reproduced with permission [177] Copyright 2016, Jeong et al. **c** The CRC-on-chip (image courtesy of Emulate, Inc.) is composed of two channels: at the top, the epithelial channel (1), hosting epithelial and CRC cells (3); at the bottom, the endothelial channel (2), hosting HUVEC cells (4). The two channels are divided by a porous membrane (5). Reproduced with permission [174]. Copy-right Strelez et al., 2021. **d** Metastasis-on-a-chip (MOC). To provide an equal flow into all device chambers, the media was perfused from the colorectal compartment and then bifurcated twice to the endothelial (E), lung (Lu), and liver (Li) constructs. Reproduced with permission [135]. Copyright 2019, John Wiley and Sons

A vascularized micro-tumor (VMT) device composed of three tissue chambers was co-cultured with different human cell types: endothelial colony-forming cell-derived endothelial cells (ECFC-EC), normal lung fibroblasts (NHLF), and colorectal cancer cells (HCT116 and SW480) (Fig. 6A) [172]. Once the cells were seeded in each tissue compartment, they were exposed to a physiological flow. This mechanical stimulus led to complex self-organization from day 5 in culture. To better characterize this innovative platform, transcriptomic analysis was performed on HCT116 cells grown in the VMT, implanted as a xenograft tumor, and cultured in the conventional 2D culture system. The results showed that gene expression of HCT116 cells from the VMT closely resembled that grown in vivo while differing from the same cells cultured in the 2D system. Indeed, several pathways were found to be enriched in the VMT and xenograft-derived cells compared to 2D monocultures, such as MAPK signaling, PI3K-Akt signaling, and microsatellite instability. Furthermore, the same comparison was performed to evaluate the response to FOLFOX (5-fluorouracil, leucovorin, and oxaliplatin), the first-line treatment for CLM patients [176]. HCT116 and SW480 cells derived from VMT or xenograft tumors showed a significant reduction in drug sensitivity compared to the cells grown in 2D. These findings suggest that the VMT system can recapitulate the in vivo CRC features, such as tumor drug response. Due to the increasing attention to the TME and its relationship with tumor progression and response to drug treatments, the interaction between CRC cells and fibroblasts was studied in a microfluidic device fabricated with seven channels: three to host colorectal adenocarcinoma cell line (HT-29) and normal colon fibroblasts (CCD-18Co), and four to provide nutrients with the medium (Fig. 6B) [177]. In this device, a significant increase in HT-29 spheroid size was observed when co-cultured with CCD-18Co compared with the monoculture spheroids suggesting the growth-promoting role of fibroblasts for tumor cells. At the same time, CCD-18Co showed an increase in the levels of αSMA and F-actin in the co-culture with tumor cells, highlighting the established crosstalk between the two cell types. Furthermore, the treatment with paclitaxel revealed that tumor cells in co-culture with fibroblasts were less sensitive to this drug treatment compared with HT-29 monoculture. This result suggests the importance of developing culture systems able to mimic the in vivo TME and its role in chemotherapeutic resistance.

### Colorectal CoCs for the metastatic behavior

Intense efforts were focused on reproducing CoC models to investigate the metastatic process of CRC cells and their dissemination to the metastatic site. To this scope, a CRC-on-chip was developed by incorporating key features of the TME (e.g., shear force mimicking peristalsis) in a two compartments device. Intestinal epithelial cells (Caco2, C2BBe1) were seeded in the upper chamber and left to generate a complete functional barrier. The lower chamber hosted endothelial cells (HUVEC). After a few days in culture, CRC cells (HC116 or HT29) were injected into the intestinal compartments and exposed to constant flow and stretch conditions, which led to the formation of CRC cell clusters on the top of the 3D structure of the intestinal cells (Fig. 6C) [174]. HCT116 cells showed a significant invasion ability compared to HT29 cells and several metabolic pathways were identified to be greatly enriched in the most invasive cell type. Furthermore, these cells were more heterogeneous in the endothelial chamber compared to the same cells on 2D plastic. In particular, the invasive characteristics of HCT116 were found to be pronounced when a fluid flow, mimicking the physiological peristalsis, was applied to the device or in the presence of cancer-associated fibroblasts. These results showed the fundamental role of each TME component in the behavior of tumor cells. A multi-organs-on-chip was developed to elucidate the mechanisms of cells spreading to different organs. The metastasis-on-chip (MOC) was designed to host the primary tumor construct and three target tissues (liver, lung, and endothelial compartments) (Fig. 6D) [135]. After ten days in culture, the cancer HTC116 cells, originally seeded in the CRC chamber, started to disseminate in the circulating flow reaching the downstream target tissues and showing a different localization between lung and liver sites. In the lung, HCT116 cells were mainly found around lung cells, while they showed higher engraftment in the liver site. Therefore, the tumor cell dissemination process and the phenomena underlying the preferential metastatic site could be understood thanks to the reproduction of a more in vivo-like environment in the multi-organs-on-chip model.

### Colorectal CoCs’ advantages and limitations

In summary, the described CRC chips represent a superior alternative to the current in vitro models. In vivo-like responses are obtained using the material and data from CRC organ-on-chip platforms [172, 176]. TME features are successfully reproduced to study how the tumor grows and reacts to pharmacological treatments [177]. Moreover, the study of the factors driving metastatic behaviors is also possible thanks to the implementation of such models [135, 174]. Notwithstanding, some important TME features are still missing, like the immune component [172, 177]. Another limit is the use of immortalized cell lines instead of patient-derived cells [172]. Indeed, the use of patient-derived cell populations is the best way to represent the heterogeneity of human cancer biology.

## Liver cancer

### Framework

Primary liver cancer is the sixth most diagnosed cancer worldwide, accounting for almost 900.000 new cases per year [178]. Hepatocellular carcinoma (HCC) and intrahepatic cholangiocarcinoma (iCCA) represent the two major histological types, resulting in 75% and 15% of all liver cases, respectively [178, 179]. Primary liver cancers are characterized by poor 5-year survival rates and few therapeutic strategies are available. Indeed, surgery still represents the main curative option for these patients [140, 180]. In the last years, intense efforts were dedicated to developing cutting-edge culture systems to mimic in vivo liver tumors and elucidate the molecular mechanisms.

### CoCs to study the liver primary tumor

**Fig. 7:**
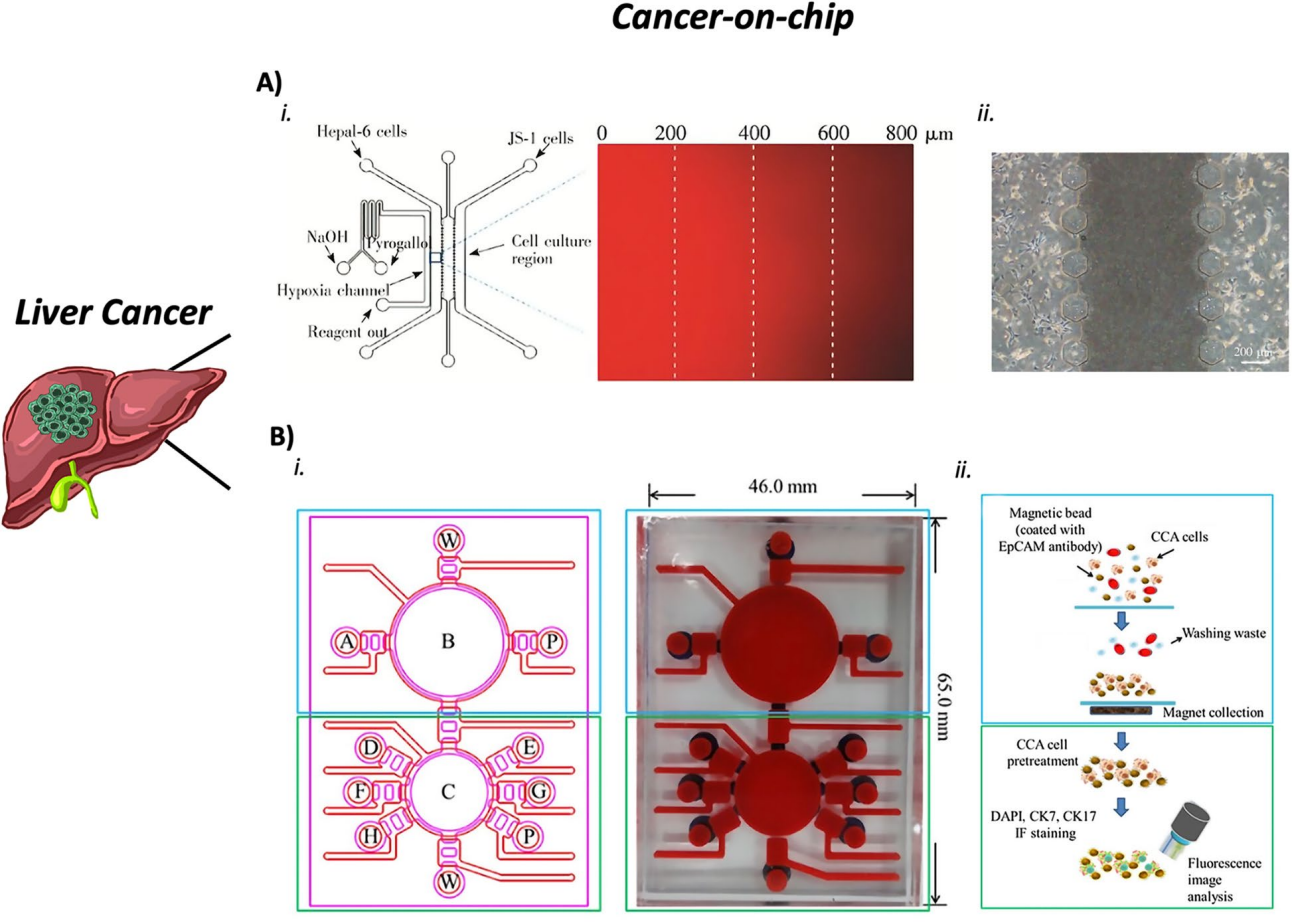
Examples of liver cancer-on-chips. **a** Schematic design of the microfluidic device. (i) The left channel was used to generate the hypoxic gradient (red fluorescent image), flanked by the culture compartment composed of three adjacent channels. (ii) Image of the co-culture compartments. Reproduced from [182] with permission from the Chinese Journal of Analytical Chemistry. **b** Cholangiocarcinoma-on-chip to detect the CTCs in the human bile. (i) Image of the chip with its compartments. Upper module: A, sample loading chamber; B, membrane-type micromixers/micropumps; P, PBS chamber; W, waste outlet. Lower module: C, membrane-type micromixers/micropumps; D, paraformaldehyde chamber; E, Triton X-100 chamber; F - G, first and secondary antibody chambers, respectively; H, DAPI/Hoechst stain chamber; P, PBS chamber; W, waste outlet. (ii) Schematic representation of the cell capture, washing, collection, and immunofluorescence (IF) staining and analysis on-chip. Reproduced from [183] Copyright Hung et al., 2017

A biomimetic liver tumor-on-chip was designed by seeding HepG2 cells, an HCC immortalized cell line, in a decellularized liver matrix enriched with gelatin methacryloyl (GelMa). This structure closely mimics the 3D complexity of the hepatic microenvironment, thanks to the presence of essential ECM proteins, growth factors, shear stress, and matrix stiffness [181]. This perfusion-based platform observed a dose-dependent response after treatment with acetaminophen and sorafenib. This result evidences that specific matrix proteins are needed to better emulate cancer biophysical properties and have an accurate platform for drug screening. A microfluidic platform was co-cultured with hepatoma cells (Hepa1-6) and hepatic stellate cells (JS-1) to study the role of hypoxia in the anticancer effect of paclitaxel (PTX) and tirapazamine (TPZ) in a more reliable microenvironment (Fig. 7A). Hepa1-6 and JS-1 cells showed a decrease in the viability after treatment with both drugs in monoculture and normoxic conditions. Instead, increased drug resistance was observed when Hepa1-6 cells were co-cultured with JS-1 cells. This effect was enhanced after PTX treatment in the hypoxic condition compared to the normoxic one. These results suggested that the activation of hepatic stellate cells could interfere with the resistance to PTX of the hepatoma cells in hypoxia and co-culture conditions [182]. This liver tumor-on-chip model demonstrated its ability to replicate the tumor niche and represents a useful platform for drug screening. Only one microfluidic system is reported in the literature for the study of CCA. This system was developed as a diagnostic tool to detect the circulating tumor cells (CTCs) in human bile (Fig. 7B) [183]. In this micrometric platform, composed of different modules, the cells isolated from the human bile are loaded and incubated with magnetic beads targeted against the EpCAM, an epithelial cell molecule, to isolate the cellular complexes (upper module of the chip). Subsequently, an immunofluorescence staining is performed with two specific anti-cytokeratin for CCA cells and, finally, the CTCs are detected and quantified in the sample (lower module of the chip). This different use of microfluidic technology underlines its potential role in the medical and biological fields.

### Liver CoCs’ advantages and limitations

In conclusion, on the one hand, liver tumor-on-chips prove to have clear advantages concerning conventional in vitro models. Biophysical properties and TME features can be recapitulated in these platforms providing better results in terms of drug screening [181, 182]. On the other hand, even if models that reproduce the CCA tumors are still undiscovered, applications studying the characteristics of these cancer cells start to appear in the scenario of microfluidic systems [183]. In general, studies that focus on elucidating metastatic behavior are still absent and more efforts are necessary to better understand and replicate the main TME components of this cancer type.

## Cancer-on-chip: pros & cons and future perspectives

**Fig. 8:**
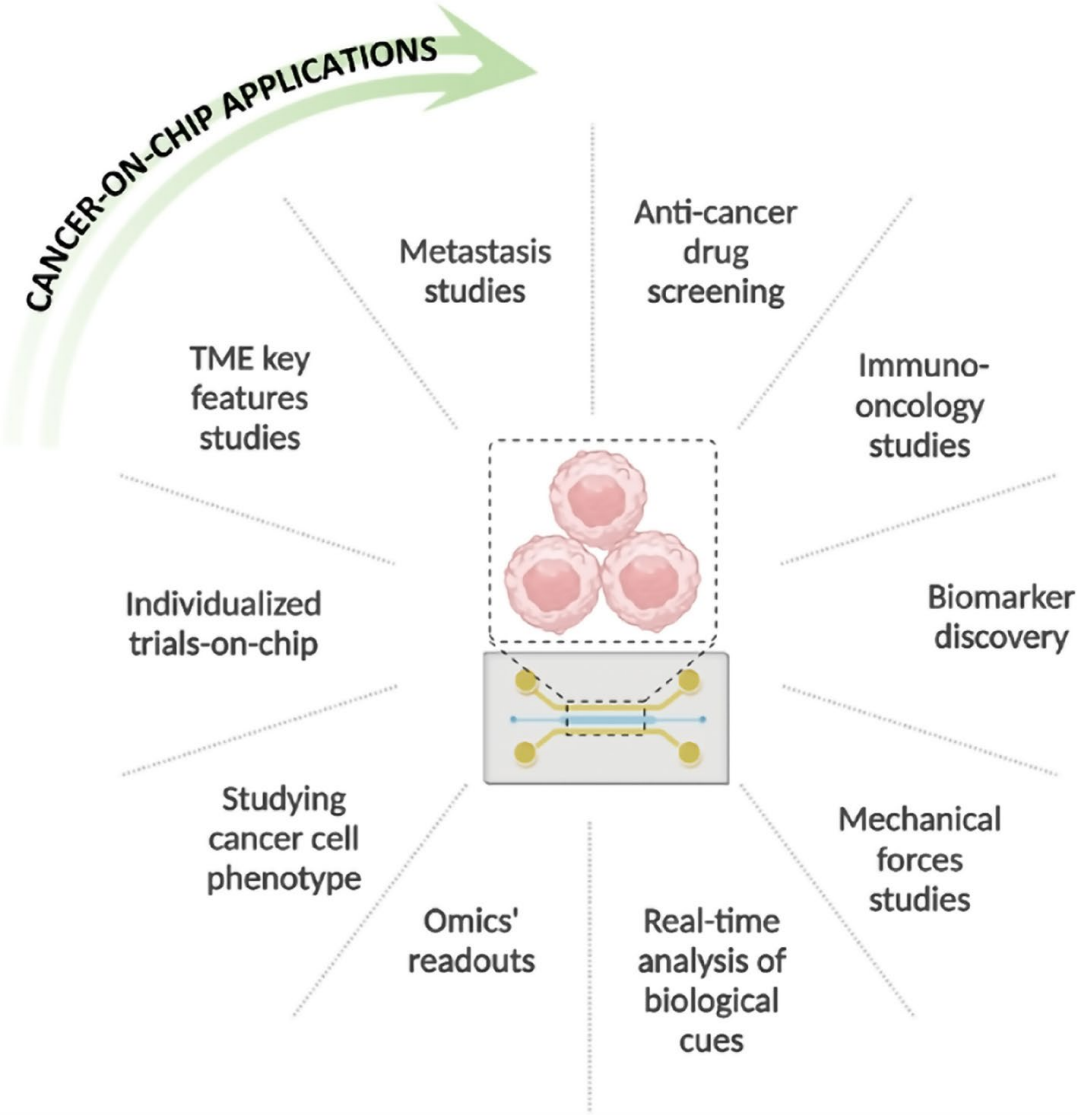
Overview of the possible cancer-on-chips applications. In general, these microfluidic devices can be used to study all cancer features and stages, to perform anti-cancer drug screening in terms of safety and efficacy, and to implement personalized medicine using patient-derived cells

Cancer-on-chip has been identified as a promising technology for studying the environment and the development of different cancers. It is becoming a possible powerful tool for different oncology applications (Fig. 8).

**Fig. 9:**
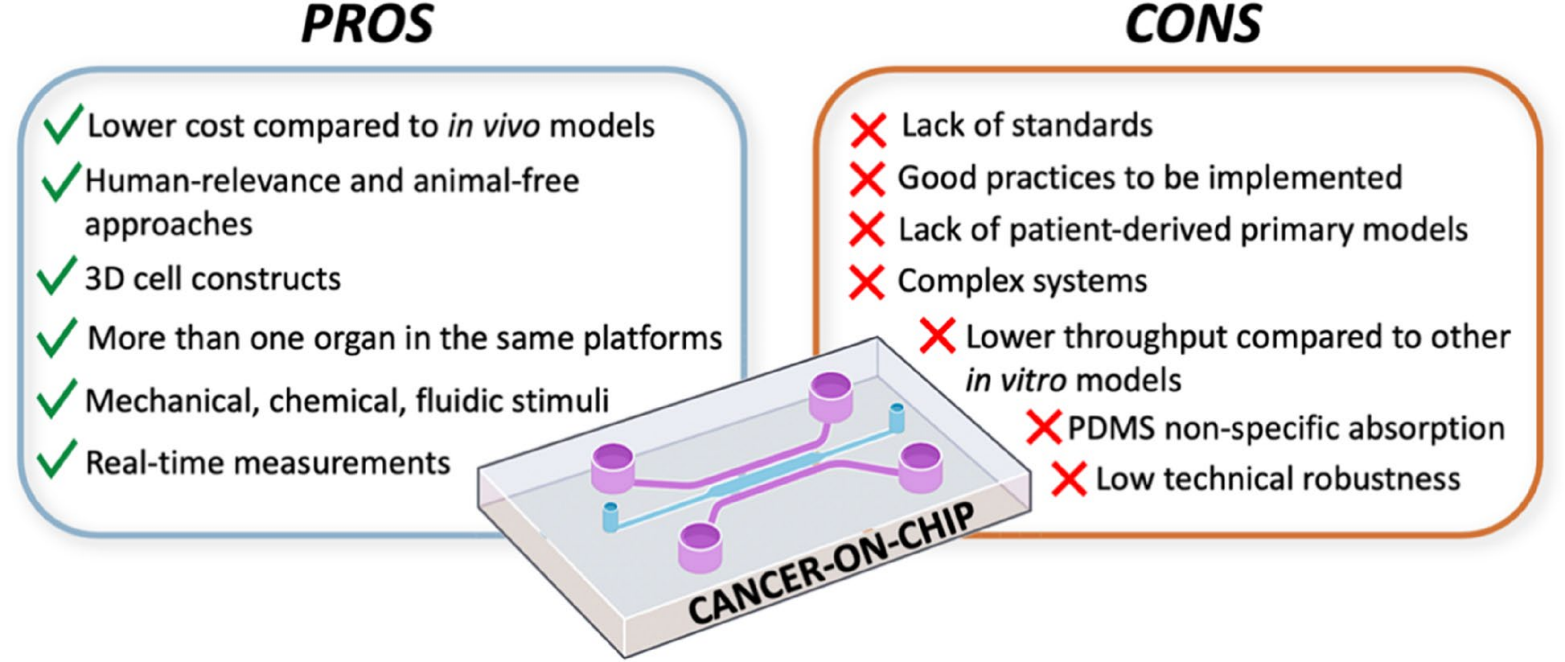
Summary of pros and cons of cancer-on-chips. Many pros are identified for the cancer-on-chip technology when compared to the conventional in vitro and in vivo models. However, acceptance of cancer-on-chip as a pre-clinical tool has several drawbacks that must be solved

These tiny devices can mimic the physiology and the pathophysiology of a target human organ, resembling human body conditions [151]. Compared to the in vivo models, the lower cost, and the possibility to have animal-free approaches should lead to extensive use of these devices, especially in the study of cancer and the possible therapies [184]. These models are considered superior to the 2D in vitro counterparts [185]. In the organ-on-chips, the implemented cell constructs are often in three dimensions, better recapitulating the cell–cell interactions by regulating key important factors such as nutrients, cytokines, and hormones. Anyway, most of the developed cancer-on-chip models still rely on the use of cell lines. Indeed, the implementation of primary cells, especially patient-derived, could be an important step to obtain a superior reproduction of the in vivo conditions. Different organs and tissues can be represented within the same platform, having a better model to study a specific phenomenon or structure [136]. The microenvironment could also be recapitulated with the flow key component. Indeed, important mechanical and chemical stimuli are generated thanks to the movement of the medium inside the designed channels, like shear stress and specific factors’ gradients, thus reproducing the in vivo conditions. The implementation of the flow and the perfused endothelial-lined vessels allow the study of several processes such as cancer cell intravasation, extravasation, and dissemination [132, 186]. Moreover, drug delivery studies are better modeled than in conventional in vitro systems thanks to the samples collected from compartments dedicated to a specific organ [143]. In the OoCs, sensors with different outputs (e.g., temperature, pH, and oxygenation) can be added to control the microenvironment and perform measurements in real-time [187]. For example, sensors have been implemented for the control and measurement of physical features, such as flow [188], temperature [189], and pH [190]. Specific sensors have been used for cancer-on-chips: an electrical cell-substrate impedance sensing (ECIS) was implemented for the monitoring of single cancer cells [191]; an electrical biosensor based on nano roughened poly(methyl methacrylate) (PMMA) was used to detect the metastatic cells [192]; and a surface plasmon resonance imaging (SPRI)-impedance sensor was applied to analyze the status of living cancer cells in real-time [193]. On one hand, this versatility represents a strong driver in the adoption of the technology, and it is essential to have trustworthy and robust organ-on-chip models. On the other hand, such sensor implementation makes organ-on-chip complex systems often not easily exploitable by people without the required expertise. In general, OoCs are more difficult to implement than other 3D models, like spheroids, which usually show a higher throughput [194]. PDMS is the most widely used material for organ-on-chip production due to its high biocompatibility, transparency, and oxygen permeability. However, the major drawback of PDMS is its nonspecific absorption of small hydrophobic molecules, including some drugs [195]. Therefore, new materials should be developed and implemented for organ-on-chip production by retaining the same important characteristics of PDMS, in terms of biocompatibility and optical clarity, but with low or null drug absorption. Solutions can be found using for example additives [196], coatings [197, 198], and completely other materials [199–201]. High pressure is put on the users to find materials that fit the purpose and the aim of the device’s application. For this reason, several manuals have appeared [202, 203] to guide users to make a more informed and correct choice. Technical robustness is another challenge that should be overcome. The small scale and the high complexity of these systems make them sensible to simple factors, like bubbles, that can impair the interplay of the implemented controls and features with the loss of organ-on-chip functionality [204]. Studies have been performed trying to prevent the formation of bubbles by providing hints about channel structuring and characteristics [205] or strategies to efficiently remove the bubbles [206]. Finally, organ-on-chip devices should be manufactured according to Good Manufacturing Practices (GMP), and the tests conducted following the in-force Good Laboratory Practices (GLP) and the Good In Vitro Method Practices (GIVIMP) [207]. These are the requirements to be recognized as pre-clinical tools. Indeed, even if most of the OoCs are tested for reproducibility, the variability due to the user-to-user component is still difficult to control. Fabrication and cell culturing methods are developed by each user mostly without any guidance. This leads to OoCs that differ in technological and biological aspects [55]. The passage from the results obtained in the micron-scale to a possible application at the macro-scale (scalability) is therefore hampered due to the lack of standardization [55]. These limitations get worse if complex multi-organ interactions are implemented. To summarize, organ-on-chip technology is still in its young phase and several challenges must be overcome as well as improvements adopted (Fig. 9). But the technological advancement of these platforms keeps on increasing. Table 2 provides an overview of some implemented solutions to reduce the impact of the described challenges. Regarding CoCs, future development relies on personalized medicine [208]. Patient-derived cells could be directly cultured within the platform providing a precise tool to better investigate the biological mechanisms underlying cancer development and to investigate the most suitable patient-specific drug therapy during the clinical trials. However, obtaining patient-specific cells is often challenging [209]. A possible solution relies on the use of stem cells. But this cellular model shows limitations in terms of technical reprogramming, increased genetic instability, especially of the induced pluripotent stem cells (iPSC), and the highly variable (but typically low) efficiency of stem cell differentiation across cell lineages [210]. Another limitation of cancer-on-chips is the simplicity of these devices, since only the essential components are usually recapitulated, missing some important chemical and physical characteristics inherent to the TME. Help is coming from the increased awareness of cancer microenvironment physiology, which is leading to more representative and even modular devices [211]. Moreover, the development of a cancer-on-chip that reproduces the entire body (body-on-chip) or multiple organs fluidically connected is aiding in a better understanding of the cancer pathophysiology, the structure, the hidden mechanisms, and the metastatic process [212].

**Table 2:** Overview of some solutions implemented to overcome or reduce the challenges faced by organ-on-chip technology

Model	Challenges	Solution	Reference
In vitro tumor microenvironment	Complex system Lower throughput Low technical robustness PDMS nonspecific absorption	Injection molded plastic array 3D culture platform for the formation of vascularized tumor spheroids in one step	[213]
Brain metastatic microenvironment due to non‐small cell lung carcinoma	Use of cell lines	Use of patient-derived cells to reproduce the brain metastatic microenvironment	[154]
Human tissue barriers	PDMS material	Implementation of inert and optically clear borosilicate glass for chip production using PDMS just as a sealing agent	[214]
Translational organ-on-chip platform	Complex system Lack of standards	Implementation of a fluidic circuit board which enables microfluidic control of multiple components like sensors or organ-on-chip devices through an interface based on openly available standards	[215]
Monitoring of cell metabolic activity	Low throughput Lack of standards Low technical robustness	The measure of oxygen consumption rates and drug-induced metabolic shifts in an array of microfluidic devices contained within an oxygen sensor-integrated microfluidic culture plate in a microtiter plate format and industry-standard footprint	[216]

## Conclusion

The current in vitro and in vivo models show evident limitations in reproducing the complexity of the TME and studying cancer progression and metastasis. Cancer-on-chips could provide the necessary complexity to express the pathophysiology and the cell–cell crosstalk within the TME and allow for studying tumor development and progression in a more in vivo-like environment. This new technology represents an advanced and unique way to reveal underlined molecular, chemical, and cellular mechanisms with a key role in cancer progression. Furthermore, the development of these platforms using patient-specific cells could help obtain a more realistic tool to faithfully recapitulate the main characteristics of the TME. The possibility to integrate multiple organs in the same platform has the huge potential to reproduce in vitro realistic models of invasiveness and metastatic tumors, thus better mimicking the intricate pathologic conditions. However, intense efforts are needed from academic researchers, manufacturers, and regulators to push the adoption of organ-on-chip technology as an alternative to the 2D in vitro and in vivo models. Indeed, the establishment of good practices as well as shared and approved standard protocols could guarantee the fundamental required quality allowing the use of organ- and cancer-on-chip as a keystone to close the gap between the pre-clinical and clinical studies.

## Data Availability

Not applicable.

## Abbreviations

2D: Two-dimensional
3D: Three-dimensional
BBB: Blood-brain barrier
BM-NSCLC: Brain metastatic non-small cell lung carcinoma
bTME: Brain tumor microenvironment
CAFs: Cancer-associated fibroblasts
CCA: Cholangiocarcinoma
CCL2: Chemokine (C–C motif) ligand 2
CCL5: Chemokine (C–C motif) ligand 5
CCL7: Chemokine (C–C motif) ligand 7
CFTR: Cystic Fibrosis Transmembrane Conductance Regulator
CLM: Colorectal liver metastasis
CoC: Cancer-on-chip
CRC: Colorectal cancer
CSE: Cigarette smoke extracts
CTC(s): Circulating tumor cell(s)
CXCL12: Chemokine (C-X-C motif) ligand 1
dT-MOC: Ductal tumor-microenvironment-on-chip
EB(s): Embryoid body(ies)
EC(s): Endothelial cell(s)
ECFC-EC: Endothelial colony-forming cell-derived endothelial cells
ECIS: Electrical cell-substrate impedance sensing
ECM: Extracellular matrix
EGFR: Epidermal growth factor receptor
EMT: Epithelial-to-mesenchymal transition
EV(s): Extracellular vesicle(s)
EVμBR(s): Microfluidic
EV: Microbioreactor(s)
FGF: Fibroblast growth factors
FLD: Fatty liver disease
FOLFOX: 5-Fluorouracil, leucovorin, and oxaliplatin
GelMa: Gelatin methacryloyl
GIVIMP: Good In Vitro Method Practices
GLP: Good Laboratory Practices
GMP: Good Manufacturing Practices) and the
HCC: Hepatocellular carcinoma
HFL1: Human fetal lung fibroblasts
IBD: Inflammatory bowel disease
iCCA: Intrahepatic cholangiocarcinoma
IL-8: Interleukin-8
IORT: Intraoperative radiotherapy
MOC: Metastasis-on-chip
MMP2: Matrix metalloproteinases 2
MMP9: Matrix metalloproteinases 9
NanoCaCO_3_: CaCO_3_ nanoparticles
NHLF: Normal lung fibroblasts
NSCLC: Non-small cell lung cancer
OoC: Organ-on-chip
PTX: Paclitaxel
PDAC: Pancreatic ductal adenocarcinoma
PDCE(s): Pancreatic ductal epithelial cell(s)
PDGF: Platelet-derived growth factors
PDMS: Polydimethylsiloxane
pH: Power of hydrogen
PLGA: Poly(lactic-co-glycolic acid)
PMMA: Poly(methyl methacrylate)
SDF-1: Stromal cell-derived factor 1
SNS: Sympathetic nervous system
SPRI: Surface plasmon resonance imaging
TEER: Transepithelial/endothelial resistance
TGFβ: Transforming growth factor beta
TKI: Tyrosine kinase inhibitor
TME: Tumor microenvironment
TPZ: Tirapazamine
TS(s): Tumor spheroid(s)
UV: Ultraviolet (light)
VMT(s): Vascularized micro-tumor(s)
μ-CCD: Microfluidic hanging drop-based spheroid co-culture device
